# Clinical characteristics and prognosis analysis of patients with de novo *ASXL1* ‐mutated AML treated with the C‐HUNAN‐AML‐15 protocol: A multicenter study by the South China Pediatric AML Collaborative Group

**DOI:** 10.1002/cam4.6005

**Published:** 2023-05-03

**Authors:** Minhui Zeng, Keke Chen, Xin Tian, Runying Zou, Xiaoqin Feng, Chunfu Li, Jian Li, Mincui Zheng, Huirong Mai, Lihua Yang, Yingyi He, Honggui Xu, Hong Wen, Xiangling He

**Affiliations:** ^1^ Department of Hematology and Oncology Children's Medical Center, Hunan Provincial People's Hospital/First Hospital of Hunan Normal University Changsha China; ^2^ Department of Pediatrics Southern Medical University/Nanfang Hospital Guangzhou China; ^3^ Nanfang‐Chunfu Children's Institute of Hematology & Oncology TaiXin Hospital Dongguan China; ^4^ Department of Hematology, Fujian Institute of Hematology Fujian Medical University Union Hospital Fuzhou China; ^5^ Hematology and Oncology Hunan Children's Hospital Changsha China; ^6^ Department of Hematology Shenzhen Children's Hospital Shenzhen China; ^7^ Zhujiang Hospital of Southern Medical University Guangzhou China; ^8^ Department of Pediatric Hematology/Oncology Guangzhou Women and Children's Medical Center Guangzhou China; ^9^ Department of Pediatric Hematology/Oncology Sun Yat‐sen Memorial Hospital Guangzhou China; ^10^ Department of Pediatric Hematology/Oncology The First Affiliated Hospital of Xiamen University Xiamen Fujian People's Republic of China

**Keywords:** AML, *ASXL1* mutation, pediatrics, prognosis, transplantation

## Abstract

**Background:**

*ASXL1* mutation is an independent prognostic factor in adult acute myeloid leukemia (AML), but its effect on the prognosis of pediatric AML is poorly understood.

**Aims:**

This study aimed to investigate the clinical characteristics and prognostic factors of *ASXL1*‐mutant pediatric AML from a large Chinese multicenter cohort.

**Methods:**

A total of 584 pediatric patients with newly diagnosed AML from 10 centers in South China were enrolled. The exon 13 of ASXL1 was amplified by polymerase chain reaction (PCR), and then analyzed the mutation status of the locus. (n = 59 for ASXL1‐mut group, n = 487 for ASXL1‐wt group).

**Results:**

*ASXL1* mutations were found in 10.81% of all patients with AML. A complex karyotype was significantly less common in the *ASXL1*‐mut AML group than in the *ASXL1*‐wt group (1.7% vs. 11.9%, *p* = 0.013). Furthermore, *TET2* or *TP53* mutations were predominantly found in the *ASXL1*+ group (*p* = 0.003 and 0.023, respectively). The 5‐year overall survival (OS) and event‐free survival (EFS) of the total cohort were 76.9% and 69.9%. In *ASXL1*‐mut AML patients, a white blood cell (WBC) count ≥50 × 10^9^/L had significantly poorer 5‐year OS and EFS than a WBC count <50 × 10^9^/L (78.0% vs. 44.6%, *p* = 0.001; 74.8% vs. 44.6%, *p* = 0.003, respectively), while receiving hematopoietic stem cell transplantation (HSCT) had a higher 5‐year OS and EFS (84.5% vs. 48.5%, *p* = 0.024; 79.5% vs. 49.3%, *p* = 0.047, respectively). In the multivariate Cox regression analysis, patients with high‐risk AML undergoing HSCT tended to have a better 5‐year OS and EFS than those receiving chemotherapy as a consolidation (HR = 0.168 and 0.260, both *p* < 0.001), and WBC count ≥50 × 10^9^/L or failure to achieve complete response after the first course were independent adverse predictors of OS and EFS (HR = 1.784 and 1.870, *p* = 0.042 and 0.018; HR = 3.242 and 3.235, both *p* < 0.001).

**Conclusion:**

The C‐HUANA‐AML‐15 protocol is a well‐tolerated and effective in the treatment of pediatric AML. *ASXL1* mutation is not an independent adverse prognosis predictor for survival in AML, whereas *ASXL1*‐mut patients tend to have a poor prognosis if WBC count ≥50 × 10^9^/L, but they can benefit from HSCT.

## 1 INTRODUCTION

Pediatric acute myeloid leukemia (AML) is a heterogeneous group of diseases classified based on lineage, genetics, and morphology. Although AML accounts for only 15%–20% of children with acute leukemia, the overall survival (OS) rate is significantly poorer than that of acute lymphoblastic leukemia (ALL), and the relapse rate is as high as 34%–38%.^1^, ^2^ However, in recent years, with the advent of cytogenetic and molecular biology tests to provide a basis for risk stratification of pediatric AML and timely initiation of appropriate treatment, the survival rate of pediatric AML has been improved. A meta‐analysis reported that 6%–30% *ASXL1* mutation were found in AML cases, a gene located in the chromosome region 20q11 that encodes a protein of the polycomb group and trithorax complex family to maintain gene expression homeostasis.^3^, ^4^ Xia's study^5^ revealed a new mechanism by which *ASXL1* mutations promote cancer, in myeloid neoplasm, the ASXL1 mutant protein, although still able to bind to BAP1, has lost its ability to interact with FOXK1/K2, resulting in an impaired function that regulates the growth of leukemia cells. Many studies have shown that the *ASXL1* mutation is an adverse predictor of adult AML, whereas few studies have been conducted in pediatric AML.^6^ Due to the differences in molecular genetics between children and adults, this study aimed to retrospectively analyze the clinical characteristics and prognosis of AML in children with *ASXL1* mutation through a large multicenter cohort, so as to better guide the prognosis prediction of AML in children and deliver precise stratified treatment.

## 2 MATERIALS AND METHODS

### 2.1 Subjects and treatment

A total of 584 pediatric patients with newly diagnosed AML from 10 centers in South China from July 2014 to December 2020 were enrolled. AML was diagnosed and classified based on the World Health Organization (WHO 2016) classification,^7^ but not include the patients with acute promyelocytic leukemia, trisomy 21 syndrome, antecedent myelodysplastic syndrome, or secondary AML. In this cohort study, 38 patients with incomplete follow‐up data who abandoned treatment or were transferred before completing induction chemotherapy were excluded, and the remaining 546 patients were evaluated in the study. We divided AML patients with and without the *ASXL1* mutation into the *ASXL1* mutation type (*ASXL1*‐mut) and ASXL1 wild‐type (*ASXL1*‐wt) groups, respectively. Additionally, we divided the groups into high risk, intermediate risk and low risk according to the protocol risk stratification criteria (Figure S1); the high‐risk group consisted of 207 patients. Clinical data were collected from all 546 pediatric patients, and cell morphology, flow cytometry, molecular analyses, and cytogenetics were performed to compare their clinical characteristics and analyze their prognostic factors.

The C‐HUANAN‐AML‐15 protocol was used on all patients (Figure S2),^8^ and intermediate‐risk and high‐risk patients who had human leukocyte antigen (HLA)‐matched donors were advised to undergo hematopoietic stem cell transplantation (HSCT). This study was approved by the ethics committees of all 10 centers and all patients provided written informed consent according to the Declaration of Helsinki to participate in the present study.

### 2.2 Laboratory methods

DNA was extracted from peripheral blood or bone marrow specimens of the children using DNA extraction kits, and the DNA concentration was determined according to the NanoDrop 2000 spectrophotometer operating standard operating procedures, requiring a sample DNA concentration of >20 ng/μL. The exon 12 of ASXL1 was amplified by polymerase chain reaction, the reaction included 95°C for 2 min, followed by 35 cycles of 95°C for 30 s, 61°C for 30 s, and 72°C for 1 min, and then sequenced in both forward and reverse directions by using Fw‐ASXL1‐Ex12 5′‐AGGTCAGATCACCCAGTCAGTT‐3′ and Rev‐ASXL1‐Ex12 5′‐TAGCCCATCTGTGAGTCCAACTGT‐3′ at the same time.^9^, ^10^ The sequence of ASXL1 was compared with normal sequences by Mutation Surveyor software to analyze the mutation status of the locus. According to the molecular detection results, 59 pediatric patients presented with *ASXL1* mutations and were included in the *ASXL1*‐mut group and the remaining 487 cases were included in the *ASXL1*‐wt group in this study.

Determination of mutations in other genes, including FLT3‐ITD, CEBPA, KIT, TP53, TET2, EVI1, and NPM1 was performed as described previously.^8^, ^11^ Karyotypes analysis by harvesting 3 mL bone marrow fluid from the newly diagnosed AML, and the metaphase chromosomes were banded by the G‐banding method. Finally, the karyotypes were described according to the international constitution of human cytogenetics (ISCN2020), and included the presence of ≥3 unrelated chromosomal abnormalities are defined as complex karyotypes.^12^, ^13^

### 2.3 Statistical analysis

Comparison of categorical variables using the Pearson's chi‐squared test or Fisher's exact test when data were sparse. OS endpoints were death or being alive at the last follow‐up, and the event‐free survival (EFS) endpoints were disease relapse or death from any cause. The cutoff date for follow‐up was April 15, 2021. The Kaplan–Meier method and log‐rank test were used to calculate the survival estimations. An unadjusted Cox proportional hazards model was adopted in univariate analyses to calculate hazard ratios, and then significant variables were enrolled in the multivariate analyses, the Cox proportional hazards model was employed to identify independent prognostic factors, and a *p* < 0.05 was considered statistically significant. SPSS 20.0 software was used to perform all statistical analyses.

## 3 RESULTS

### 3.1 Clinical characteristics of AML with *ASXL1* mutation

A total of 546 pediatric patients with AML were included in this study for clinical analysis, risk stratification according to the protocol criteria classified 207 cases (37.9%) into the high‐risk group (HSCT 113 cases), 262 cases (48.0%) into the intermediate‐risk group (HSCT 96 cases) and 115 cases (21.1%) into the low‐risk group (HSCT 31 cases). *ASXL1* mutations ware found in 10.81% (59/546) of all 546 pediatric AML, and in 28.50% (59/207) of the high‐risk group patients. As regards the clinical characteristics of the *ASXL1*‐mut AML and *ASXL1*‐wt AML patients (Table 1), we found no significant differences between the two groups in terms of age, sex, initial white blood cell (WBC) counts, FAB subtype, and the complete response (CR) rate after the second course of chemotherapy or blasts in bone >15% after the first course of chemotherapy. A complex karyotype was significantly less common in the *ASXL1‐*mut AML than in the *ASXL1*‐wt AML (1.7% vs. 11.9%, *p* = 0.013), while there was no significant difference in the distribution of other karyotypes. Furthermore, we also counted genes with a higher frequency of mutations and found that *TET2* (11.9% vs. 2.7%, *p* = 0.003) or *TP53* mutations (6.8% vs. 1.4%, *p* = 0.023) were significantly more common in the *ASXL1‐*mu*t* AML than in the *ASXL1*‐wt AML. However, the differences in the frequencies of *FLT3‐ITD* mutations, the *CEBPA* ^*dm*^ mutation, the *CEBPA* ^*sm*^ mutation, and the *C‐KIT* mutation were not significant between the two groups (Table 1).

**TABLE 1:** Clinical and genetic characteristics according to ASXL1 mutation.

	ASXL1‐mut, *n* (%)	ASXL1‐wt, *n* (%)	*p‐*value
Age (years)			
<10	46 (78.0)	380 (78.0)	0.991
≥10	13 (22.0)	107 (22.0)	
Sex			
Male	42 (71.2)	283 (58.7)	0.053
Female	17 (28.8)	204 (41.3)	
WBC, ×10^9^/L			
<50	42 (71.2)	332 (68.2)	0.638
≥50	17 (28.8)	155 (31.8)	
FAB subtype			
M0	0 (0)	12 (2.4)	0.628
M1	0 (0)	15 (3.1)	0.390
M2	25 (42.4)	159 (32.7)	0.136
M4	6 (10.2)	31 (6.4)	0.272
M5	20 (33.9)	163 (33.5)	0.948
M6	0 (0)	4 (0.8)	1.000
M7	4 (6.8)	36 (7.4)	1.000
Undifferentiated type	4 (6.8)	67 (13.8)	0.132
Cytogenetic characteristics			
t (8;21)	14 (23.7)	72 (14.8)	0.075
Inv (16)/t (16;16)	3 (5.1)	25 (5.1)	1.000
−7 or del (7q)	3 (5.1)	17 (3.5)	0.467
t (9;11)	5 (8.5)	22 (4.5)	0.198
Complex karyotype	1 (1.7)	58 (11.9)	0.013
Normal karyotype	13 (22.0)	147 (30.2)	0.227
Molecular abnormalities			
FLT3‐ITD	6 (10.2)	58 (11.9)	0.695
CEBPA^dm^‐mutation	2 (3.4)	16 (3.3)	1.000
CEBPA^sm‐^mutation	2 (3.4)	13 (2.7)	0.671
C‐KIT‐mutation	5 (8.5)	56 (11.5)	0.486
EVI1	3 (3.4)	33 (5.8)	0.786
TET2	7 (11.9)	13 (2.7)	0.003
TP53	4 (6.8)	7 (1.4)	0.023
NPM1	1 (1.7)	12 (2.5)	1.000
CR after the second course of induction			
Yes	52 (88.1)	413 (84.8)	0.736
No	4 (6.8)	36 (7.4)	
Missing	3 (5.1)	38 (7.8)	
Blast >15% in BM after the first course of induction			
Yes	0 (0)	21 (4.3)	0.266
No	58 (98.3)	458 (94.1)	
Missing	1 (1.7)	8 (1.6)	

### 3.2 Overall survival of AML

**FIGURE 1:**
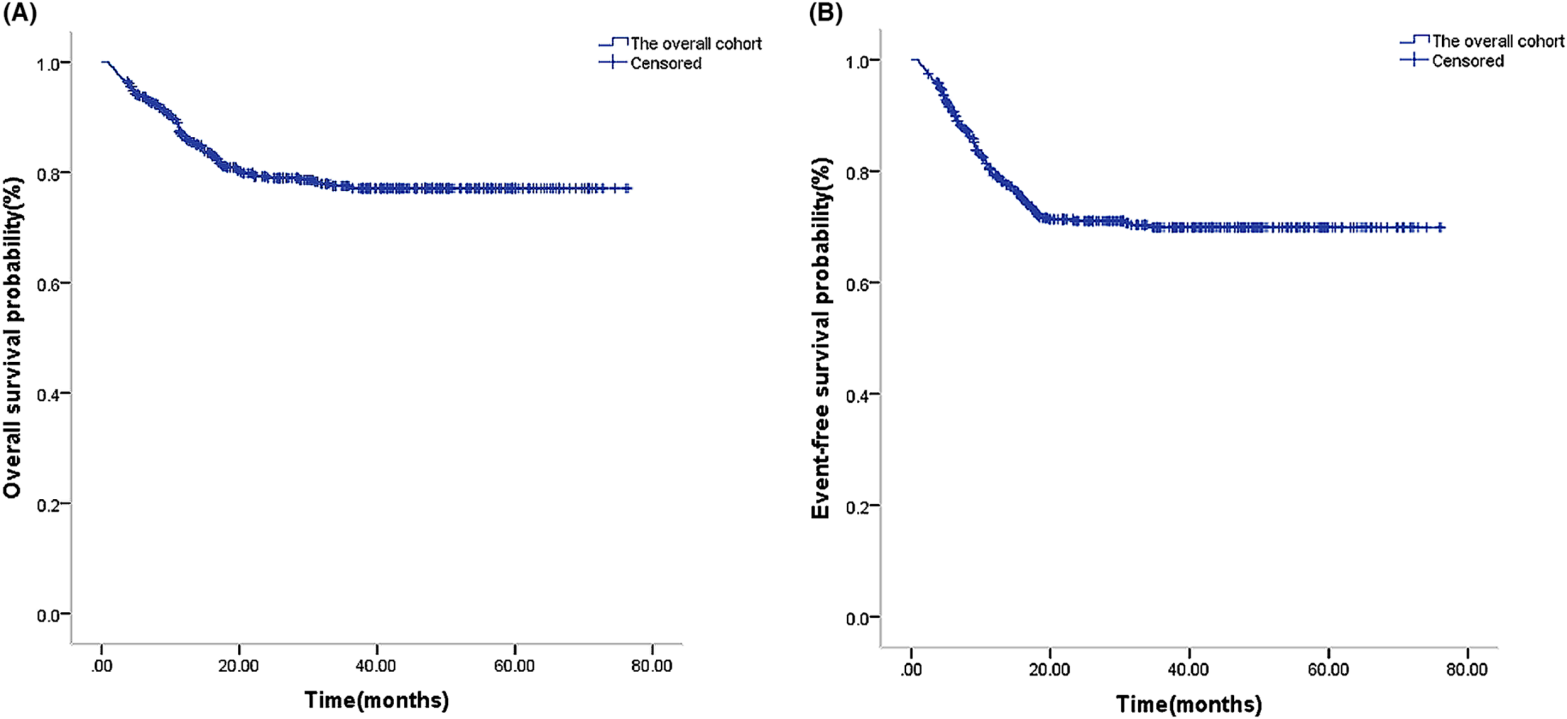
Survival outcome for the overall cohort of pediatric AML. Kaplan–Meier curve estimates for (A) overall survival (OS) (76.9%) and (B) event‐free survival (EFS) (69.9%) in the total cohort.

**FIGURE 2:**
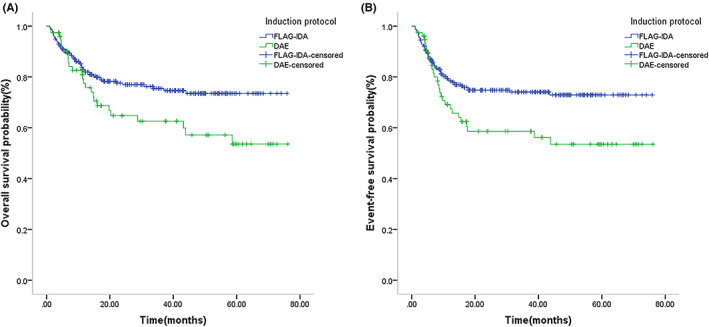
Survival outcome for patients with pediatric acute myeloid leukemia (AML) who received chemotherapy as consolidation therapy between FLAG‐IDA group or DAE group. Kaplan–Meier curve estimates for (A) overall survival (OS) (*p* = 0.039) and (B) event‐free survival (EFS) (*p* = 0.015) in the cohort between FLAG‐IDA group and DAE group in pediatric AML who received chemotherapy as consolidation therapy.

The median follow‐up time for survival of the 546 pediatric AML patients was 23.3 months (range 1–76.1 months). A total of 432 cases (79.12%) survived, and 16 (2.93%) were lost to the last follow‐up. Of the 98 deaths (17.95%), 41 were due to disease recurrence (including posttransplantation recurrence), 23 were due to a concomitant serious infection, 11 were due to transplantation‐related complications, and 23 were chemotherapy‐related deaths alone. The treatment‐related mortality with chemotherapy alone was not significantly significant than HSCT (7.5% [23/306] vs. 4.6% [11/240], *p* = 0.159). The 5‐year OS and EFS were 76.9% and 69.9% in the total cohort, respectively (Figure 1). The CR rate was 81.4% (437/537) after the first course of chemotherapy and 91.7% (463/505) after the second course of chemotherapy, with 15.8% (86/546) patients relapsing. A total of 409 cases were selected to undergo the FLAG‐IDA regimen, and the CR rate after the first course of receiving FLAG‐IDA induction was not significantly higher than the DAE induction (85.8% vs. 83.2%, *p* = 0.727); however, in the analysis of children treated with chemotherapy as consolidation therapy, the FLAG‐IDA regimen group had a significantly better 5‐year OS and EFS than the DAE group (OS: 73.5% vs. 53.6%, *p* = 0.039; EFS: 72.9% vs. 53.5%, *p* = 0.015) (Figure 2).

### 3.3 Prognosis of AML with *ASXL1* mutation

**FIGURE 3:**
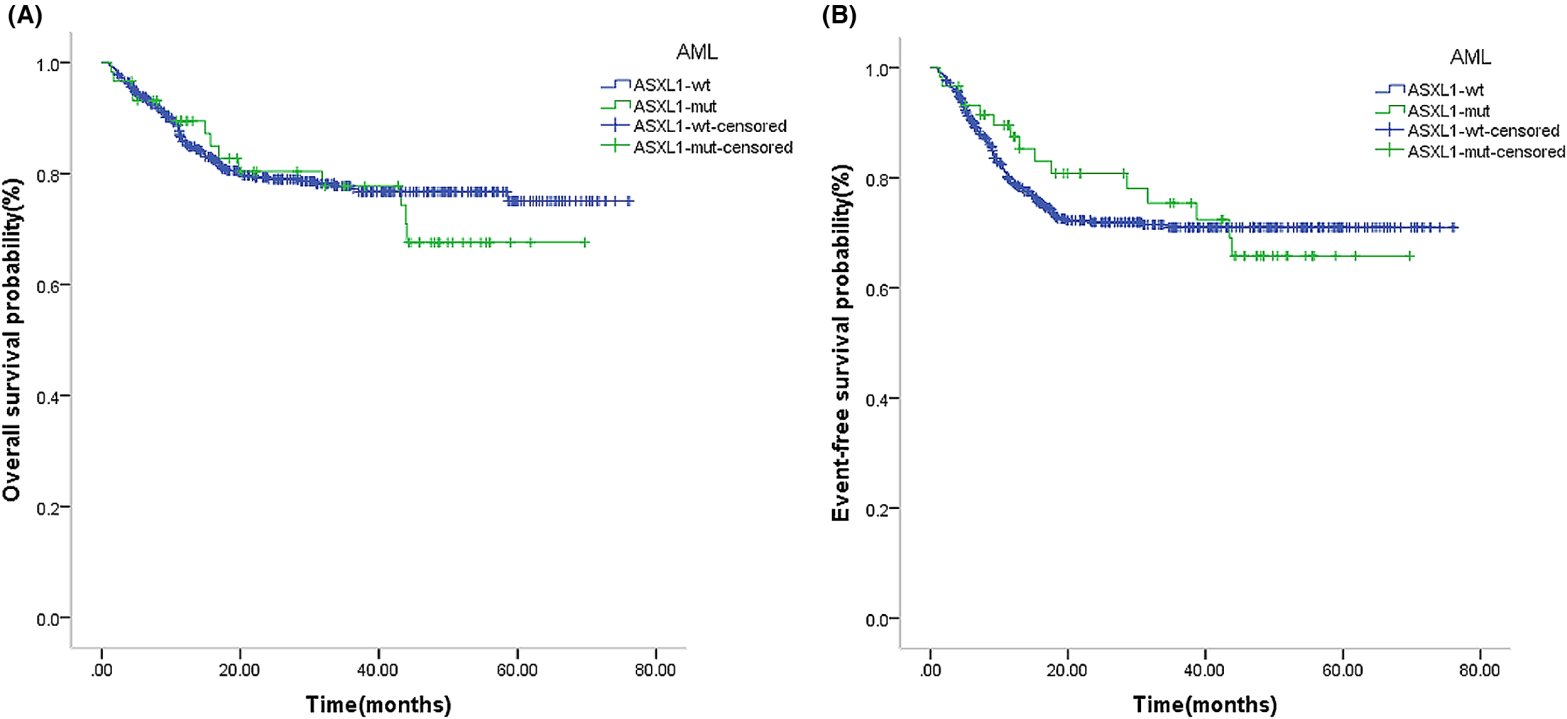
Survival outcome for pediatric AML with ASXL1 mutation. Kaplan–Meier curve estimates for (A) overall survival (OS) (*p* = 0.616) and (B) EFS (*p* = 0.766) in the total cohort between ASXL1‐mut and ASXL1‐wt AML patients.

**FIGURE 4:**
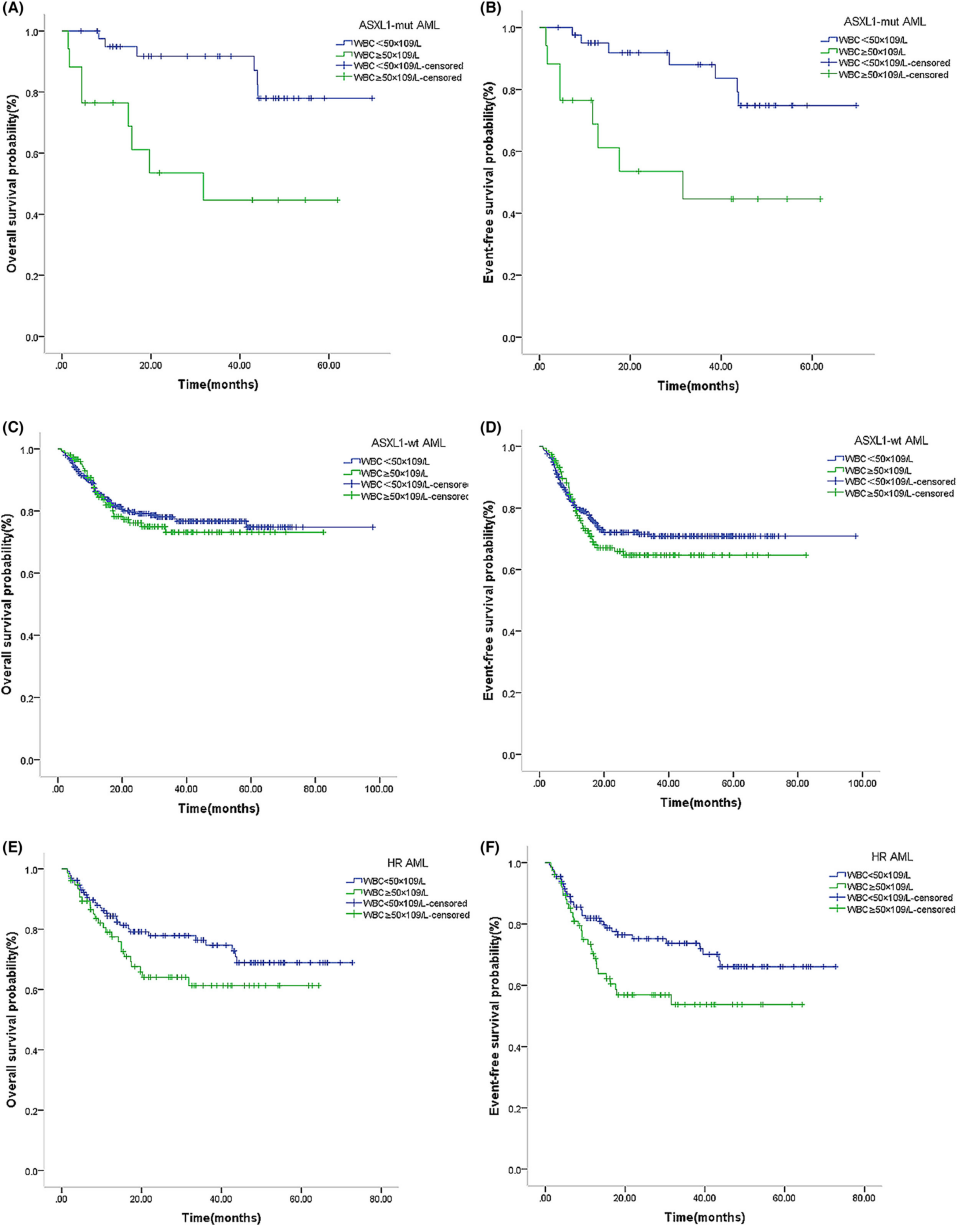
Survival outcome for ASXL1‐mut or ASXL1‐wt AML or HR AML patients with WBC ≥50 × 10^9^/L or WBC <50 × 10^9^/L. Kaplan–Meier curve estimates for (A) overall survival (OS) (*p* = 0.001) and (B) EFS (*p* = 0.003) in the cohort between the ASXL1‐mut AML patients with WBC ≥50 × 10^9^/L and WBC <50 × 10^9^/L. Kaplan–Meier curve estimates for (C) OS (*p* = 0.609) and (D) EFS (*p* = 0.318) in the cohort between the ASXL1‐wt AML patients with WBC ≥50 × 10^9^/L and WBC <50 × 10^9^/L. Kaplan‐ Meier curve estimates for (E) OS (*p* = 0.112) and (F) EFS (*p* = 0.027) in the cohort between the HR AML patients with WBC ≥50 × 10^9^/L and WBC <50 × 10^9^/L.

Seven *ASXL1*‐mut AML patients had a *TET2* mutation, all of whom survived after consolidation chemotherapy, and four cases had a *TP53* mutation (among whom one underwent HSCT after relapse, two underwent HSCT after CR1, and one case received chemotherapy as consolidation), all of whom survived. The *ASXL1*‐mut AML (59 cases) had inferior 5‐year OS and EFS than the *ASXL1*‐wt AML (487 cases), but the differences were not statistically significant (OS: 67.6% vs. 75.0%, *p* = 0.616; EFS: 65.8% vs. 71.0%, *p* = 0.766) (Figure 3). Among *ASXL1*‐mut AML patients, WBC ≥50 × 10^9^/L (nine cases) had a significant inferior 5‐year OS and EFS than WBC <50 × 10^9^/L (50 cases) (OS: 78.0% vs. 44.6%, *p* = 0.001; EFS: 74.8% vs. 44.6%, *p* = 0.003) (Figure 4). ASXL1‐wt AML patients in combination with WBC ≥50 × 10^9^/L (150 cases) predicted a trend of inferior 5‐year OS and EFS than WBC <50 × 10^9^/L (337 cases) (OS: 73.2% vs. 74.8%, *p* = 0.609; EFS: 64.8% vs. 70.8%, *p* = 0.318) (Figure 4).

**FIGURE 5:**
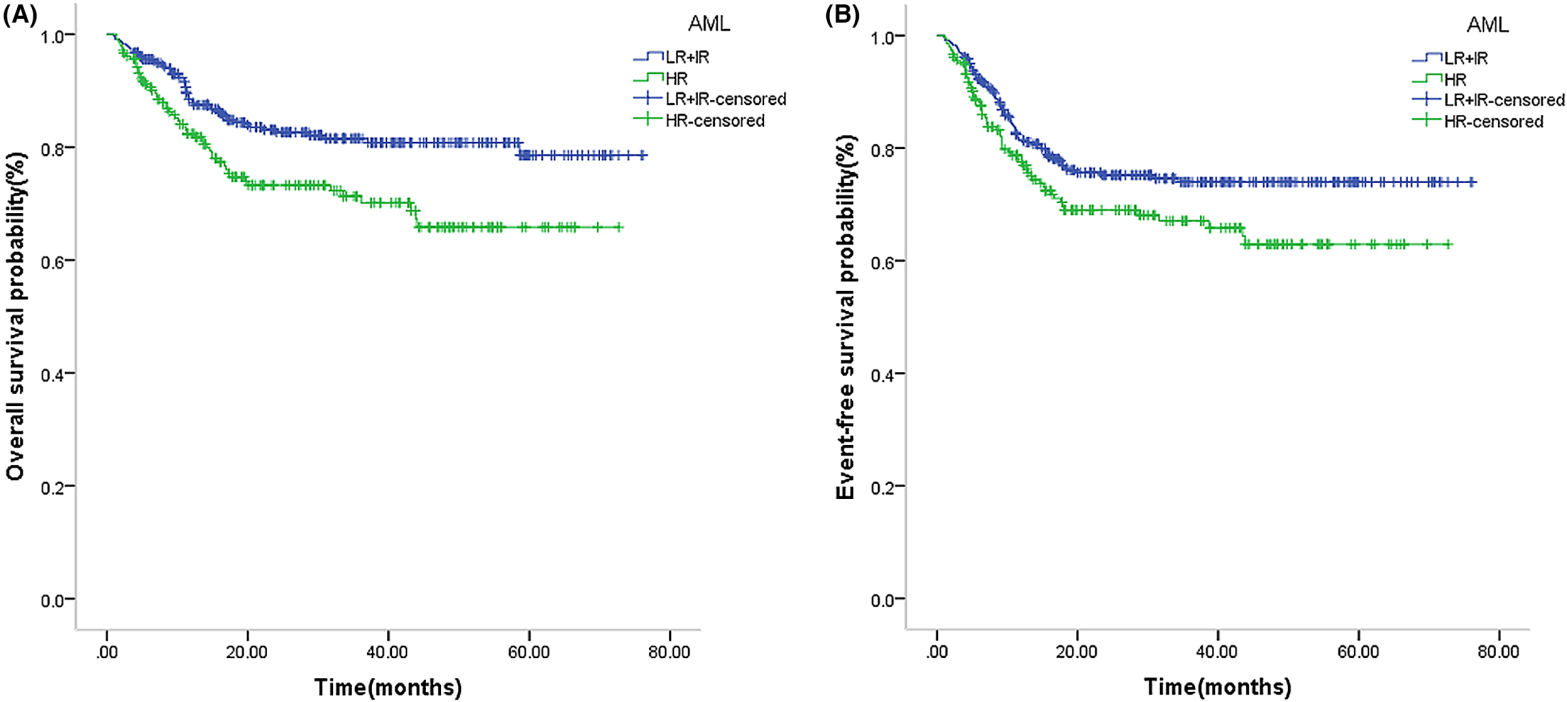
Survival outcome for HR patients and LR+IR patients in pediatric AML. Kaplan–Meier curve estimates for (A) overall survival (OS) (*p* = 0.003) and (B) EFS (*p* = 0.037) in the cohort between HR and LR+IR patients based on the protocol risk stratification criteria.

The high‐risk group (207 cases) had significantly poorer 5‐year OS and EFS than the low‐ and intermediate‐risk group (339 cases) (OS: 65.8% vs. 78.5%, *p* = 0.003; EFS: 62.9% vs. 74.0%, *p* = 0.037), with statistically significant differences (Figure 5). The HR AML combination with WBC ≥50 × 10^9^/L (76 cases) had a significant inferior 5‐year EFS (53.7% vs. 66.1%, *p* = 0.027), excluding OS (61.3% vs. 68.9%, *p* = 0.112) (Figure 4).

### 3.4 Survival of children who underwent HSCT in the high‐risk and *ASXL1‐*mut groups

**FIGURE 6:**
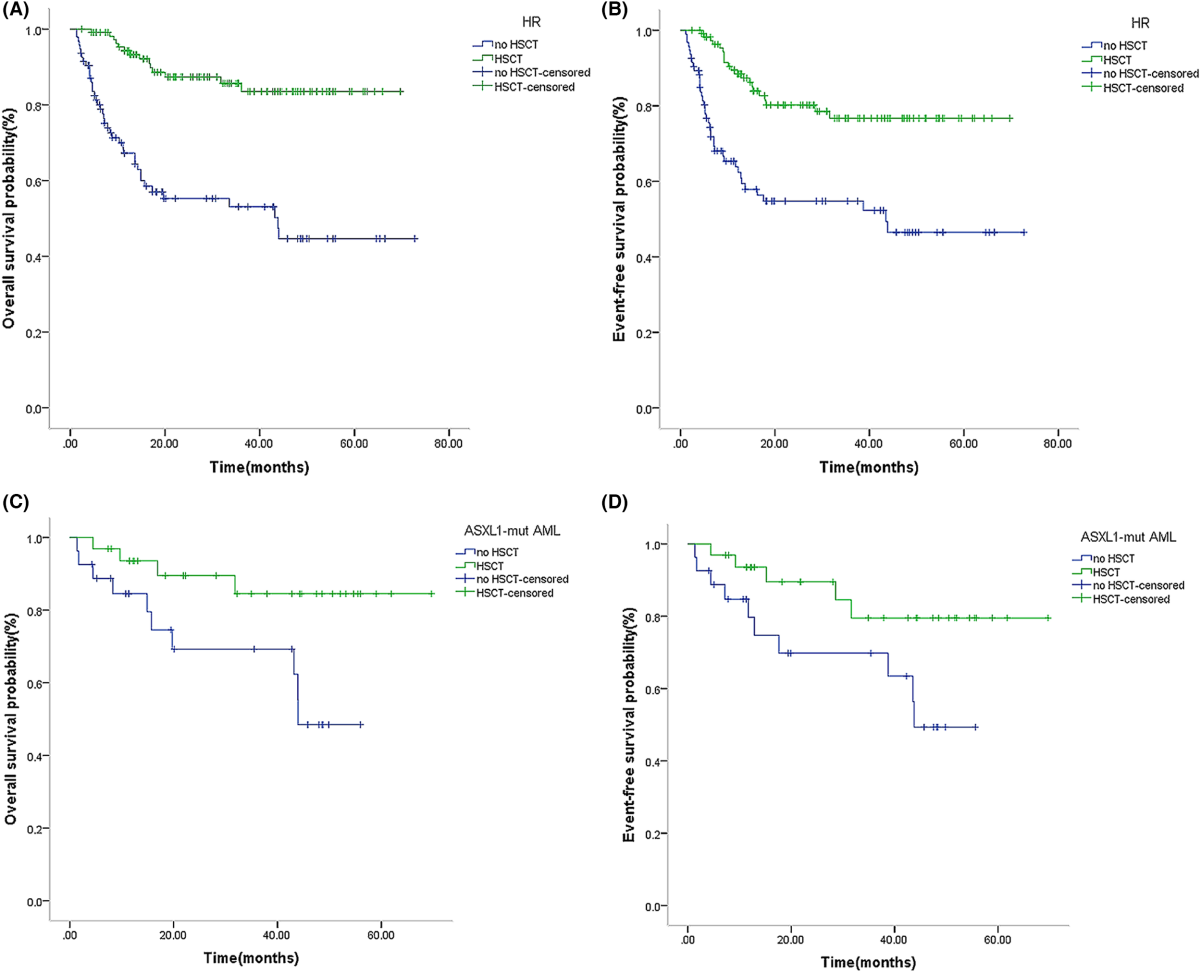
Survival outcome for pediatric AML who underwent HSCT or received chemotherapy as consolidation therapy in the HR group and the ASXL1‐mut group. Kaplan–Meier curve estimates for (A) OS (*p* < 0.001) and (B) EFS (*p* < 0.001) in the cohort who underwent HSCT or received chemotherapy as consolidation therapy in HR group. Kaplan–Meier curves for(C) OS (*p* = 0.024) and (D) EFS (*p* = 0.047) of ASXL1‐mut AML who underwent HSCT or received chemotherapy as consolidation therapy.

According to the C‐HUANAN‐AML‐15 treatment protocol used in this study, HSCT is recommended for high‐risk pediatric AML during the consolidation phase; however, some pediatric patients did not undergo transplantation but chose to continue chemotherapy as consolidation therapy. The transplantation group accounted for 54.6% (113/207), and we found that the high‐risk patients who underwent HSCT had significantly higher 5‐year OS and EFS than the patients receiving chemotherapy as consolidation (OS: 83.5% vs. 44.6%, *p* < 0.001; EFS: 76.7% vs. 46.5%; *p* < 0.001) (Figure 6). All *ASXL1*‐mut group patients were included in the high‐risk group, and 32 cases (54.2%) underwent transplanted in the *ASXL1‐*mut group, who underwent HSCT had a significantly higher 5‐year OS and EFS than the patients receiving chemotherapy as consolidation (OS: 84.5% vs. 48.5%, *p* = 0.024; EFS: 79.5% vs. 49.3%, *p* = 0.047) (Figure 6).

### 3.5 Univariate and multifactorial analyses for assessing risk factors in high‐risk pediatric AML

In this study, a univariate analysis was used to evaluate risk factors for high‐risk pediatric AML including the *FLT3‐ITD* mutation, the *ASXL1* mutation, complex karyotype, HSCT, age, sex, WBC, and induction protocol (DAE) and failure to achieve CR after the first course (Table 2). We found that HSCT was a favorable predictor of improved OS and EFS in high‐risk AML (HR = 0.208 and 0.314, both *p* < 0.001), and failure to achieve CR after the first course of chemotherapy was associated with significantly adverse OS and EFS in high‐risk pediatric AML (all HR >1, *p* < 0.001). Furthermore, WBC ≥50 × 10^9^/L was an independent prognostic factor for EFS (HR = 1.829, *p* < 0.019), but not OS. The multivariate Cox analysis was performed to evaluate independent prognostic factors, the AML patients in high‐risk who underwent HSCT with a tendential survival benefit compared to chemotherapy as a consolidation and was a favorable independent influence factor (HR = 0.168 and 0.260, both *p* < 0.001); however, WBC ≥50 × 10^9^/L or failure to achieve CR after the first course were independent adverse predictors of OS and EFS (HR = 1.784 and 1.870, *p* = 0.042 and 0.018, respectively; HR = 3.242 and 3.235, both *p* < 0.001, respectively) (Table 2).

**TABLE 2:** Univariate and multivariate analyses of patients in HR AML.

Case (*n*)		OS			EFS	
Univariate analysis	HR	95% CI	*p‐*value	HR	95% CI	*p‐*value
ASXL1 mutation	0.741	0.402–1.365	0.336	0.664	0.370–1.192	0.170
FLT3‐ITD mutation	1.178	0.669–2.075	0.570	1.348	0.799–2.274	0.263
Complex karyotype	0.932	0.498–1.742	0.825	0.803	0.435–1.483	0.483
HSCT	0.208	0.113–0.383	<0.001	0.314	0.185–0.533	<0.001
Age (≥10 years)	1.075	0.576–2.008	0.820	1.793	0.610–1.958	0.766
Sex (male)	1.174	0.661–2.086	0.584	1.218	0.708–2.195	0.475
WBC (≥50 × 10^9^/L)	1.587	0.928–2.715	0.091	1.829	1.105–3.328	0.019
No CR after first course	3.389	1.945–5.905	<0.001	3.394	2.006–5.742	<0.001
Induction protocol (DAE)	1.114	0.605–2.051	0.729	1.030	0.575–1.847	0.920

Multivariate analysis	HR	95% CI	*p‐*value	HR	95% CI	*p‐*value
HSCT	0.168	0.089–0.318	<0.001	0.260	0.150–0.450	<0.001
WBC (≥50 × 10^9^/L)	1.784	1.022–3.113	0.042	1.870	1.114–3.140	0.018
No CR after first course	3.242	1.671–6.292	0.001	3.235	1.751–5.977	<0.001

Abbreviations: AML, acute myeloid leukemia; CR, complete response; HSCT, hematopoietic stem cell transplantation.

## 4 DISCUSSION

In recent years, the OS rate of AML according to the International Collaborative Clinical Research Group of Advanced Treatment Centers has reached 60%–70%, but this remains far lower than that of ALL.^2^, ^14^, ^15^ Although the *ASXL1* mutation is often associated with a poor prognosis in adult AML, this is still controversial. The *ASXL1* gene is involved in epigenetic regulation, and its mutation usually leads to C‐terminal truncation of proteins upstream of PHD, which encourages gene dysfunction and triggers the occurrence of AML^16^; therefore, the *ASXL1* mutation is not only involved in the pathogenesis of AML but is also associated with its prognosis. We carried out this clinical study to examine whether the *ASXL1* mutation can be included in a prognostic evaluation system for AML and to provide a theoretical basis for delivering individualized treatment of pediatric AML.

A total of 584 pediatric patients with newly diagnosed AML from 10 centers in South China were enrolled, and 546 patients were finally enrolled in the study. A modified C‐HUANA‐AML‐15 regimen, with the MRC AML15 protocol as the backbone, was used as the unified treatment protocol for pediatric AML.^17^, ^18^ Our results showed that the overall 5‐year OS following C‐HUANA‐AML‐15 treatment was 76.9%, which is not significantly higher than that of the protocols of other collaborative groups,^19^ and the chemotherapy‐related mortality was low. In this study, our collaborative group mainly used FLAG‐IDA induction as a treatment regimen, the CR rate after first of course was 85.8%, and the survival rate was significantly better than the DAE induction, indicating that the FLAG‐IDA induction is more effective in the treatment of Chinese pediatric AML, and that it is an effective induction treatment that provides deeper remission and a better prognosis. Previous studies have indicated that FLAG‐IDA induction is a well‐tolerated and effective regimen for the treatment of relapsed refractory AML, particularly those with epigenetic alterations.^20^

*ASXL1* mutations were found in 10.81% of the 546 pediatric AML patients, lower than the 17% reported in adults and may be related to the lower frequency of ASXL1 mutations in people younger than 40 years of age.^11^ Our study found that complex karyotypes were significantly less common in ASXL1‐mut AML than in ASXL1‐wt AML, consistent with reports stating that ASXL1 mutations were observed less frequently in AML adult patients with complex karyotypes, but there were no data of children, and larger sample sizes are needed to verify the results.^10^ However, concomitant *TET2* mutations or *TP53* mutations were more frequently found in the *ASXL1‐*mut group. *TET2* mutations are associated with DNA hypermethylation, and previous studies have indicated that they are associated with a poor prognosis in AML.^21^, ^22^, ^23^ However, it should be noted that seven patients with the *TET2* mutation in the *ASXL1*‐mut AML group survived, possibly due to the low number of patients. Even though the *TET2* mutation is not currently defined as a risk stratification indicator for AML, and larger sample sizes are needed to investigate whether *TET2* mutation should be included as an adverse risk factor of AML and whether demethylation therapy can be used in *TET2*‐mutated pediatric AML to improve its survival rate. The human TP53 gene is an important anti‐oncogene, TP53 mutations are infrequent, appearing in approximately 12.7% of AML patients.^24^, ^25^ Some studies have reported that the *TP53* mutation is associated with poor prognosis in AML,^26^ but Koji Sasaki's group found that in patients with *TP53* mutations,^11^ the coexistence of *ASXL1* mutations did not seem to affect prognosis, so a larger sample size subsequently is needed to determine their prognostic value.

In this study, the total cohort was divided into high‐risk, intermediate‐risk, and low‐risk groups according to the protocol risk stratification criteria. All ASXL1 mutant patients were included in the high‐risk group, which was subjected to univariate and multifactorial analyses. We found that HSCT had a better OS and EFS in patients with high‐risk pediatric AML, and failing to achieve CR after the first course of chemotherapy and WBC ≥50 × 10^9^/L were independent adverse predictors of a poor OS and EFS. Among children with *ASXL1*‐mut AML, WBC ≥50 × 10^9^/L was associated with significantly poorer survival than a low WBC count, the difference wasn't significant in ASXL1‐wt AML, but there was a significantly inferior 5‐year EFS in HR AML, which further confirmed that children with higher WBC counts tended to have an inferior prognosis when combined with other risk factors and can provide guidance for our clinical practice.^27^, ^28^ By comparing the patients with ASXL1‐mut AML or high‐risk AML who underwent HSCT with those who underwent chemotherapy as a consolidation, we found that HSCT improved the prognosis and provided benefit to pediatric AML with the ASXL1 mutation. This conclusion is consistent with other studies.^29^

Many previous studies have suggested that ASXL1 mutations are associated with a poor prognosis in AML, and significantly reduced the overall survival.^6^, ^30^ However, other studies have reported that ASXL1 mutations showed poor outcomes in older (≥60 years) AML and normal karyotype AML patients; otherwise, they did not have any impact on OS and disease‐free survival (DFS).^31^ Our study found that 5‐year OS and EFS were not significantly lower in pediatric AML with ASXL1 mutation than in ASXL1‐wt AML patients. Further analysis showed that 54.24% of the children with the ASXL1 mutation underwent HSCT. Only a small portion of the children in the ASXL1‐wt group underwent HSCT. Meanwhile, multivariate analyses also showed that HSCT could improve the prognosis of children, leading to the observation that the ASXL1‐mut AML patients did not show significant poor prognosis. We speculated that the ASXL1 mutation alone may not be an independent prognostic factor for AML. But, ASXL1‐mut AML patients with a higher WBC had an inferior prognosis, similar to previous reports.^27^, ^28^ Therefore, when ASXL1‐mut AML patients present with a high leukocyte count, transplantation may be a timely and effective treatment, providing a strong basis for the individualized and precise treatment of AML.

Another report defined the risk factors for adverse OS and EFS in AML patients with ASXL1 mutation as high risk factors, including WBC count ≥50 × 10^9^/L, age ≥60 years, RUNX1 mutation, FLT3‐ITD mutation, and AML1‐ETO fusion gene deletion. Their study suggests that risk factors for ASXL1‐mut AML contribute to adverse outcomes in these patients,^32^ which is similar to our findings. However, due to the small number of patients with ASXL1‐mut AML combined with other gene mutations in this study, survival analysis could not be performed. In the future, reliable conclusions need to be made by further long‐term follow‐up or increasing the sample size.

A limitation of this study is lack of treatment randomization and low adherence to HSCT recommendations.

In conclusion, the C‐HUANA‐AML‐15 protocol is well‐tolerated and effective in the treatment of pediatric AML, and HSCT can improve the prognosis of high‐risk and ASXL1‐mut pediatric AML. ASXL1 mutation is not an independent risk factor for AML in children, so it remains to be verified whether ASXL1 mutation can be used as a standalone high‐risk factor for AML.

## AUTHOR CONTRIBUTIONS

**Minhui Zeng:** Conceptualization (lead); data curation (lead); formal analysis (lead); investigation (lead); methodology (lead); resources (lead); validation (lead); visualization (lead); writing – original draft (lead); writing – review and editing (lead). **Keke Chen:** Data curation (equal); formal analysis (equal); investigation (equal); methodology (equal); resources (equal); validation (equal); visualization (equal); writing – original draft (equal); writing – review and editing (equal). **Xin Tian:** Data curation (supporting); formal analysis (supporting); investigation (supporting); methodology (supporting); resources (supporting). **Runying Zou:** Data curation (supporting); formal analysis (supporting); investigation (supporting); resources (supporting); validation (supporting). **Xiaoqin Feng:** Data curation (supporting); investigation (supporting); resources (supporting). **Chunfu Li:** Data curation (supporting); investigation (supporting); resources (supporting). **Jian Li:** Data curation (supporting); resources (supporting). **Mincui Zheng:** Data curation (supporting); resources (supporting). **Huirong Mai:** Data curation (supporting); resources (supporting). **Li‐Hua Yang:** Data curation (supporting); resources (supporting). **Yingyi He:** Data curation (supporting); resources (supporting). **Hong‐Gui Xu:** Data curation (supporting); resources (supporting). **Hong Wen:** Data curation (supporting); resources (supporting). **Xiangling He:** Conceptualization (equal); data curation (equal); formal analysis (equal); investigation (equal); methodology (equal); project administration (lead); resources (equal); validation (equal); visualization (equal); writing – review and editing (equal).

## Supporting information


Figure S1.

Figure S2.

## Data Availability

The data that support the findings of this study are available from the corresponding author upon reasonable request.

## References

[1] Madhusoodhan PP , Carroll WL , Bhatla T . Progress and prospects in pediatric leukemia. Curr Probl Pediatr Adolesc Health Care. 2016;46(7):229‐241. 27283082 10.1016/j.cppeds.2016.04.003

[2] Kim H . Treatments for children and adolescents with AML. Blood Res. 2020;55(S1):S5‐S13. 32719170 10.5045/br.2020.S002 PMC7386885

[3] Prats‐Martín C , Burillo‐Sanz S , Morales‐Camacho RM , et al. ASXL1 mutation as a surrogate marker in acute myeloid leukemia with myelodysplasia‐related changes and normal karyotype. Cancer Med. 2020;9(11):3637‐3646. 32216059 10.1002/cam4.2947 PMC7286456

[4] Asada S , Fujino T , Goyama S , Kitamura T . The role of ASXL1 in hematopoiesis and myeloid malignancies. Cell Mol Life Sci. 2019;76(13):2511‐2523. 30927018 10.1007/s00018-019-03084-7 PMC11105736

[5] Xia YK , Zeng YR , Zhang ML , et al. Tumor‐derived neomorphic mutations in ASXL1 impairs the BAP1‐ASXL1‐FOXK1/K2 transcription network. Protein Cell. 2021;12(7):557‐577. 32683582 10.1007/s13238-020-00754-2 PMC8225741

[6] Schnittger S , Eder C , Jeromin S , et al. ASXL1 exon 12 mutations are frequent in AML with intermediate risk karyotype and are independently associated with an adverse outcome. Leukemia. 2013;27(1):82‐91. 23018865 10.1038/leu.2012.262

[7] Papaemmanuil E , Gerstung M , Bullinger L , et al. Genomic classification and prognosis in acute myeloid leukemia. N Engl J Med. 2016;374(23):2209‐2221. 27276561 10.1056/NEJMoa1516192 PMC4979995

[8] Zheng Y , Huang Y , Le S , et al. High EVI1 expression predicts adverse outcomes in children with De novo acute myeloid leukemia. Front Oncol. 2021;11:712747. 34589425 10.3389/fonc.2021.712747 PMC8474639

[9] Chou WC , Huang HH , Hou HA , et al. Distinct clinical and biological features of de novo acute myeloid leukemia with additional sex comb‐like 1 (ASXL1) mutations. Blood. 2010;116(20):4086‐4094. 20693432 10.1182/blood-2010-05-283291

[10] Kakosaiou K , Panitsas F , Daraki A , et al. ASXL1 mutations in AML are associated with specific clinical and cytogenetic characteristics. Leuk Lymphoma. 2018;59(10):2439‐2446. 29411666 10.1080/10428194.2018.1433298

[11] Sasaki K , Kanagal‐Shamanna R , Montalban‐Bravo G , et al. Impact of the variant allele frequency of ASXL1, DNMT3A, JAK2, TET2, TP53, and NPM1 on the outcomes of patients with newly diagnosed acute myeloid leukemia. Cancer. 2020;126(4):765‐774. 31742675 10.1002/cncr.32566

[12] Liehr T . International System for Human Cytogenetic or Cytogenomic Nomenclature (ISCN): some thoughts. Cytogenet Genome Res. 2021;161(5):223‐224. 34407536 10.1159/000516654

[13] Mrózek K , Eisfeld AK , Kohlschmidt J , et al. Complex karyotype in de novo acute myeloid leukemia: typical and atypical subtypes differ molecularly and clinically. Leukemia. 2019;33(7):1620‐1634. 30737482 10.1038/s41375-019-0390-3 PMC6609457

[14] Ward E , DeSantis C , Robbins A , Kohler B , Jemal A . Childhood and adolescent cancer statistics, 2014. CA Cancer J Clin. 2014;64(2):83‐103. 24488779 10.3322/caac.21219

[15] Kiem Hao T , Van Ha C , Huu Son N , Nhu HP . Long‐term outcome of childhood acute myeloid leukemia: a 10‐year retrospective cohort study. Pediatr Rep. 2020;12(1):8486. 32308973 10.4081/pr.2020.8486 PMC7160853

[16] Gelsi‐Boyer V , Brecqueville M , Devillier R , Murati A , Mozziconacci MJ , Birnbaum D . Mutations in ASXL1 are associated with poor prognosis across the spectrum of malignant myeloid diseases. J Hematol Oncol. 2012;5:12. 22436456 10.1186/1756-8722-5-12 PMC3355025

[17] Russell N , Hills R , Kjeldsen L , Dennis M , Burnett A . Treatment intensification with FLAG‐Ida may improve disease control in younger patients with secondary acute myeloid leukaemia: long‐term follow up of the MRC AML15 trial. Br J Haematol. 2022;196(6):1344‐1347. 34904225 10.1111/bjh.17974

[18] Burnett AK , Russell NH , Hills RK , et al. Optimization of chemotherapy for younger patients with acute myeloid leukemia: results of the medical research council AML15 trial. J Clin Oncol. 2013;31(27):3360‐3368. 23940227 10.1200/JCO.2012.47.4874

[19] Rasche M , Zimmermann M , Borschel L , et al. Successes and challenges in the treatment of pediatric acute myeloid leukemia: a retrospective analysis of the AML‐BFM trials from 1987 to 2012. Leukemia. 2018;32(10):2167‐2177. 29550834 10.1038/s41375-018-0071-7 PMC6170392

[20] Pommert L , Schafer ES , Malvar J , et al. Decitabine and vorinostat with FLAG chemotherapy in pediatric relapsed/refractory AML: report from the therapeutic advances in childhood leukemia and lymphoma (TACL) consortium. Am J Hematol. 2022;97(5):613‐622. 35180323 10.1002/ajh.26510 PMC8986610

[21] Awada H , Nagata Y , Goyal A , et al. Invariant phenotype and molecular association of biallelic TET2 mutant myeloid neoplasia. Blood Adv. 2019;3(3):339‐349. 30709865 10.1182/bloodadvances.2018024216 PMC6373752

[22] Qu X , Zhang S , Wang S , et al. TET2 deficiency leads to stem cell factor‐dependent clonal expansion of dysfunctional erythroid progenitors. Blood. 2018;132(22):2406‐2417. 30254129 10.1182/blood-2018-05-853291 PMC6265651

[23] Gaidzik VI , Paschka P , Späth D , et al. TET2 mutations in acute myeloid leukemia (AML): results from a comprehensive genetic and clinical analysis of the AML study group. J Clin Oncol. 2012;30(12):1350‐1357. 22430270 10.1200/JCO.2011.39.2886

[24] Prokocimer M , Molchadsky A , Rotter V . Dysfunctional diversity of p53 proteins in adult acute myeloid leukemia: projections on diagnostic workup and therapy. Blood. 2017;130(6):699‐712. 28607134 10.1182/blood-2017-02-763086 PMC5659817

[25] Olivier M , Hollstein M , Hainaut P . TP53 mutations in human cancers: origins, consequences, and clinical use. Cold Spring Harb Perspect Biol. 2010;2(1):a001008. 20182602 10.1101/cshperspect.a001008 PMC2827900

[26] Short NJ , Montalban‐Bravo G , Hwang H , et al. Prognostic and therapeutic impacts of mutant TP53 variant allelic frequency in newly diagnosed acute myeloid leukemia. Blood Adv. 2020;4(22):5681‐5689. 33211826 10.1182/bloodadvances.2020003120 PMC7686900

[27] Xu LH , Wang JW , Wang Y , Yang FY . Hyperleukocytosis predicts inferior clinical outcome in pediatric acute myeloid leukemia. Hematology. 2020;25(1):507‐514. 33317436 10.1080/16078454.2020.1859169

[28] Stahl M , Shallis RM , Wei W , et al. Management of hyperleukocytosis and impact of leukapheresis among patients with acute myeloid leukemia (AML) on short‐ and long‐term clinical outcomes: a large, retrospective, multicenter, international study. Leukemia. 2020;34(12):3149‐3160. 32132655 10.1038/s41375-020-0783-3 PMC8155811

[29] Zhou L , An J , Hou C , et al. Allogeneic hematopoietic stem cell transplantation could improve the survival of acute myeloid leukemia patients with ASXL1 mutations. Hematology. 2021;26(1):340‐347. 33840380 10.1080/16078454.2021.1905356

[30] Alvarez Argote J , Dasanu CA . ASXL1 mutations in myeloid neoplasms: pathogenetic considerations, impact on clinical outcomes and survival. Curr Med Res Opin. 2018;34(5):757‐763. 28027687 10.1080/03007995.2016.1276896

[31] Lin Y , Wang Y , Zheng Y , Wang Z , Wang Y , Wang S . Clinical characteristics and prognostic study of adult acute myeloid leukemia patients with ASXL1 mutations. Hematology. 2020;25(1):446‐456. 33250015 10.1080/16078454.2020.1847801

[32] Fan Y , Liao L , Liu Y , et al. Risk factors affect accurate prognosis in ASXL1‐mutated acute myeloid leukemia. Cancer Cell Int. 2021;21(1):526. 34627254 10.1186/s12935-021-02233-y PMC8502294

