# Supplementary figures and images for: Clinical characteristics and prognosis analysis of patients with de novo ASXL1 ‐mutated AML treated with the C‐HUNAN‐AML‐15 protocol: A multicenter study by the South China Pediatric AML Collaborative Group
Source: Cancer Med. 2023 May 3;12(12):13182–92. doi: 10.1002/cam4.6005 (PMC10315855; doi:10.1002/cam4.6005)

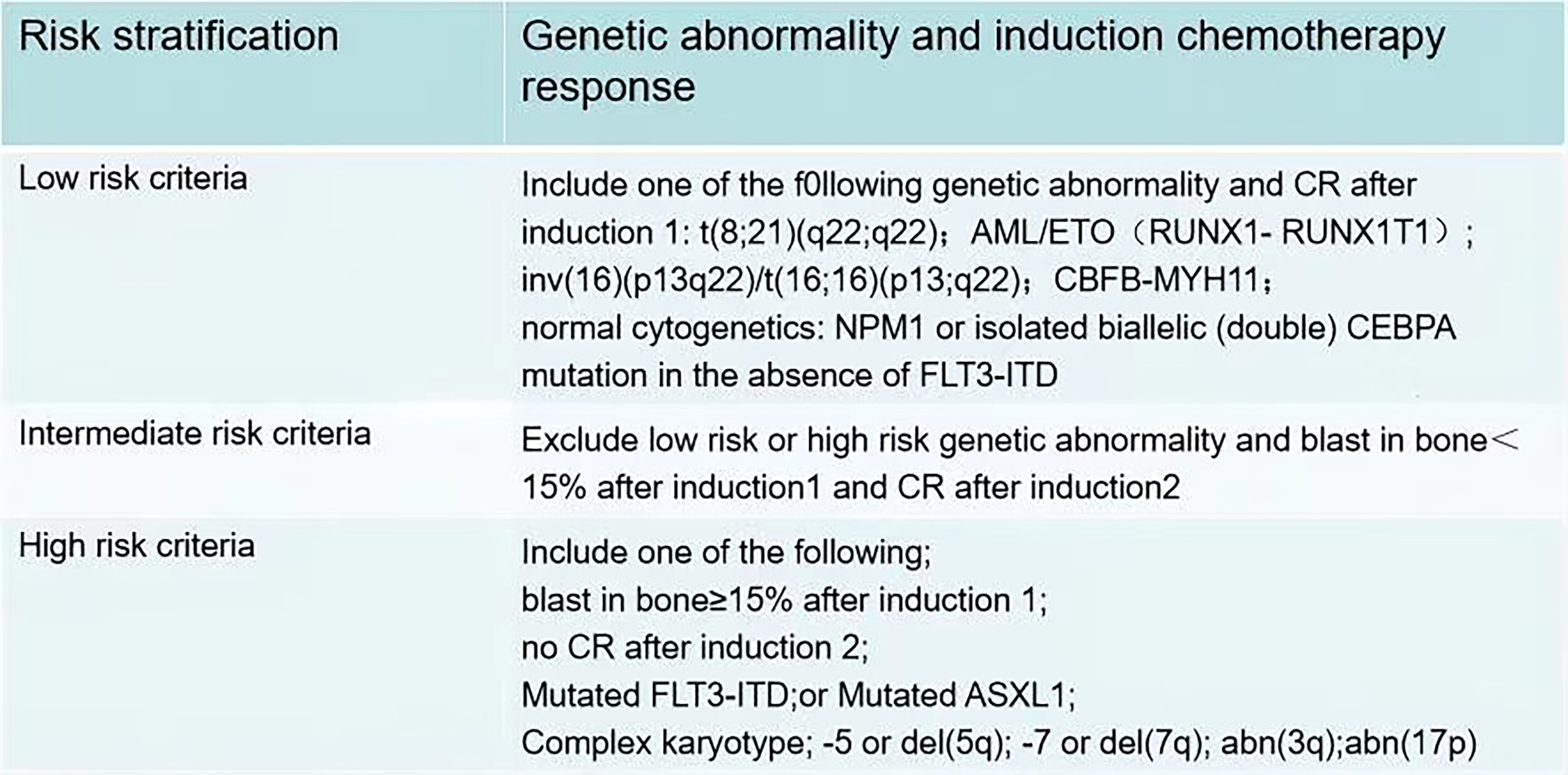

Supplement: Supplementary file 1 — Figure S1. [file CAM4-12-13182-s002.tif]

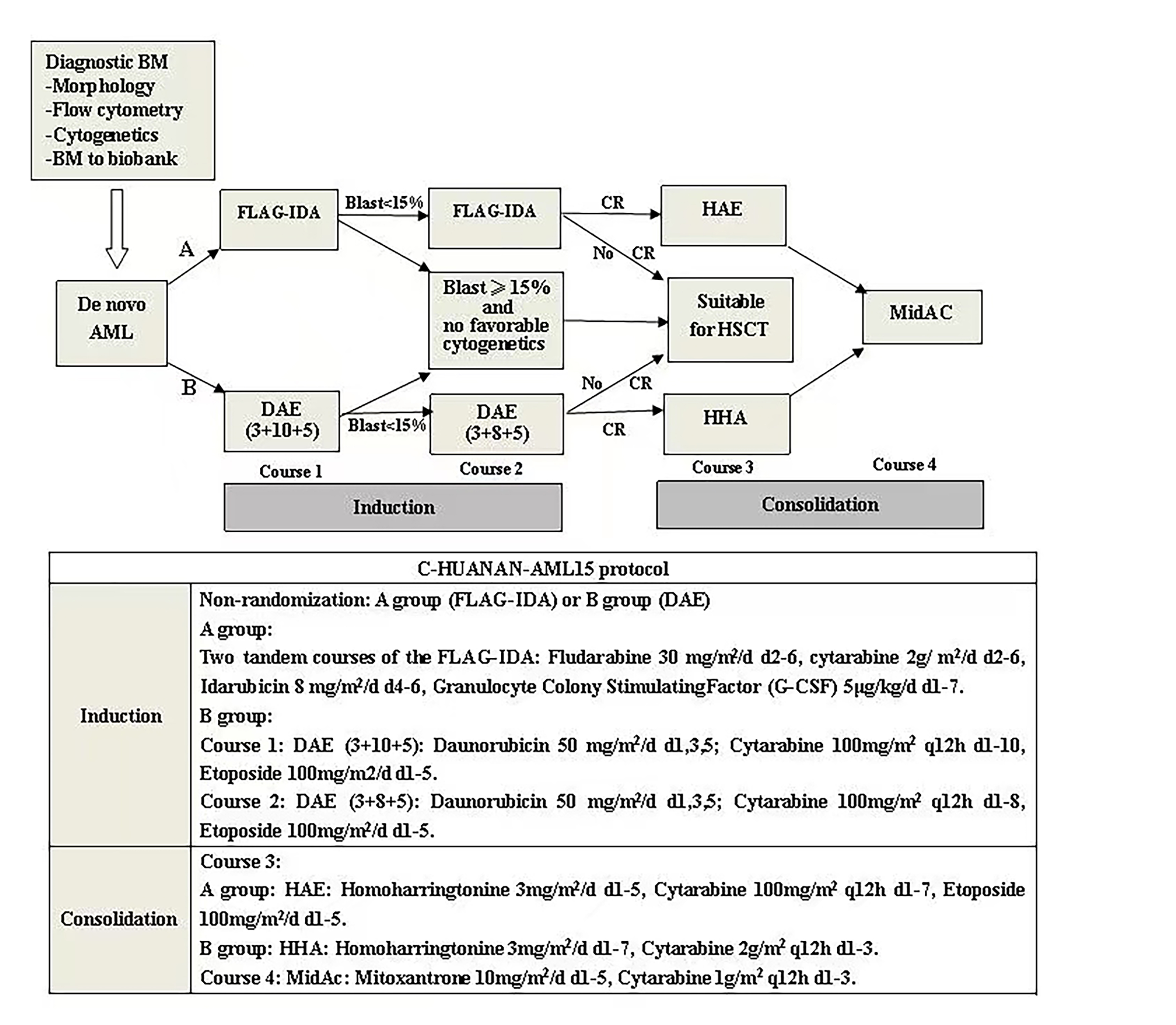

Supplement: Supplementary file 2 — Figure S2. [file CAM4-12-13182-s001.tif]
